# Primary Care Physicians' Interactions With a Novel Web-Based Active Learning Tool (The Community Fracture Capture Learning Hub): Qualitative Analysis

**DOI:** 10.2196/78472

**Published:** 2026-05-19

**Authors:** Ahmed M Fathalla, Shanton Chang, Cherie Chiang, Ralph Audehm, Alexandra Gorelik, Christopher J Yates, Steve Snow, Rahul D Barmanray, Sarah Price, Lucy Collins, John D Wark

**Affiliations:** 1 Department of Medicine, The Royal Melbourne Hospital, University of Melbourne, 300 Grattan Street, Melbourne, Victoria, 3050, Australia, 61 0383445892; 2 School of Computing and Information Systems, University of Melbourne, Melbourne, Australia; 3 Department of Diabetes and Endocrinology, Royal Melbourne Hospital, Melbourne Health, Melbourne, Australia; 4 Department of General Practice and Primary Care, University of Melbourne, Melbourne, Australia; 5 School of Public Health and Preventive Medicine, Monash University, Melbourne, Australia; 6 Praxhub, Melbourne, Australia; 7 Department of Obstetric Medicine, Royal Women's Hospital, University of Melbourne, Melbourne, Australia; 8 Department of Medicine, School of Clinical Sciences, Monash University, Melbourne, Australia

**Keywords:** community-based fracture capture learning hub, osteoporosis, virtual communities of practice, continuing professional development, primary care physicians, web-based learning platform, case-based education, evidence-based decision-making, collaborative learning, online learning, qualitative analysis

## Abstract

**Background:**

Osteoporosis poses a significant global health burden and is responsible for over 8.9 million fragility fractures annually. Despite evidence-based guidelines and treatment, a substantial care gap persists, with only a low percentage of fracture patients receiving guideline-concordant care. Primary care physicians (PCPs) are pivotal in community-based fracture prevention but face challenges in translating knowledge into practice. While hospital-based fracture liaison services are effective, their reach is limited, necessitating scalable alternatives. Virtual communities of practice and web-based learning tools offer promising avenues for PCP professional education; however, their application in osteoporosis management remains underexplored. The Community Fracture Capture (CFC) Learning Hub was developed as an interactive, case-based platform to address these gaps by enhancing PCPs’ knowledge, confidence, and engagement in osteoporosis care.

**Objective:**

The study aimed to conduct a qualitative evaluation of PCPs’ interactions with the CFC Learning Hub, focusing on barriers and facilitators of the online learning tool and exploring PCP perceptions of the program.

**Methods:**

A qualitative analysis was performed using data from 55 PCPs across four 6-week cycles of the CFC Learning Hub (May 2022-October 2023). Data sources included discussion forum comments and responses to open-ended questions in end-of-cycle evaluations. Relational content analysis was used, with 2 researchers independently coding data using semantic and latent approaches. Themes were identified through iterative discussions and validated against existing literature.

**Results:**

Four themes emerged from PCP interactions: (1) user experience–guided platform design, where participants emphasized intuitive navigation, minimized fragmented sections, and clarity of interface as critical for engagement; (2) learning-supportive course structure, highlighting the importance of explicit links between case studies and foundational knowledge, weekly summaries, and quizzes aligned with content; (3) learners’ different styles and preferences, with diverging needs for synchronous vs asynchronous learning, didactic sessions, and peer-to-peer interactions; and (4) program content, where participants requested expanded topics and postprogram refreshers.

**Conclusions:**

The CFC Learning Hub demonstrated efficacy as a specialist- and peer-to-peer–supported online learning model for PCPs, addressing osteoporosis care gaps through user-centered design, adaptable content delivery, and collaborative moderation. Key successes included resolving usability issues iteratively and accommodating diverse learning preferences. These findings underscore the potential of the Hub to enhance primary care professional education and fracture prevention. The study advocates for broader adoption of the platform to bridge osteoporosis care disparities.

## Introduction

### Background

#### Addressing the Global Burden of Osteoporosis: Challenges and Opportunities in Fracture Prevention

Osteoporosis is a major global health issue, affecting approximately 200 million people worldwide and resulting in over 8.9 million fractures annually, or 1 fracture every 3 seconds [1,2]. With the global population aging, this burden is expected to increase significantly in the coming decades, with projections suggesting an up to 310% rise in hip fractures for men and a 240% increase for women by 2050, compared to 1990 rates [3-5]. In Australia, the total annual costs of osteoporosis and osteopenia management in Australians aged ≥50 years were estimated at $4.8 billion (~US $3.4 billion) in 2023 and projected to increase to >$8.3 billion (>US $~5.9 billion) by 2033 [6]. Despite the rising prevalence and the significant morbidity, mortality, and economic burden caused by fragility fractures, there remains a substantial gap in the effective implementation of interventions aimed at fracture prevention [3,7,8]. Although diagnostic tools such as dual-energy X-ray absorptiometry (DXA) and fracture risk assessment tools such as FRAX [9,10] are available, and medications exist to reduce fracture risk, only 20%‐30% of individuals who sustain fragility fractures receive care based on established guidelines [3,6,11]. For instance, despite changes in reimbursement guidelines, there has been a plateau in subsidized bone density testing for Australians aged ≥70 years, despite the aging population [12,13].

Hospital-based fracture liaison services (FLSs) are a cost-effective model for secondary fracture prevention and represent an exemplary standard of postfracture care. They provide a multidisciplinary, patient-centered approach that systematically identifies, assesses, and manages individuals following a fragility fracture through coordinated input from multiple specialists and are internationally recognized as the most effective strategy for preventing subsequent fractures in patients with osteoporosis [14,15]. However, fractures that do not require hospital care, such as many vertebral or radial fractures, are often managed solely in primary care clinics, where patients may miss out on these services [16,17]. Additionally, recent modeling has suggested that expanding hospital-based FLS would only moderately reduce fracture incidence in older adults and at a high financial cost, underscoring the need for alternative, community-based strategies [18]. Therefore, adapting hospital-based fracture prevention efforts for use in primary care represents a rational and potentially scalable approach. Unlike hospital-based models that rely on coordinators, community-based prevention places full responsibility on primary care physicians (PCPs) to identify, investigate, and manage osteoporosis [19]. Notably, the translation of evidence into clinical practice remains low among PCPs in Australia and other countries, leading to suboptimal care for many at-risk individuals [11,20-22]. While hospital FLS programs focus on secondary prevention, PCPs are uniquely positioned to also address primary prevention and reduce long-term fracture risk [23].

#### Leveraging Virtual Communities of Practice to Bridge the Osteoporosis Care Gap in Primary Care

PCPs face significant challenges in managing multiple chronic health conditions within the constraints of limited consultation time, requiring continuous knowledge expansion to ensure evidence-based practices are applied [24,25]. As a result, there is an increasing reliance on online resources for continuing professional development [16,17,26], offering a flexible, time-efficient alternative to traditional learning methods. Social media tools and virtual communities of practice (VCoPs) have become popular platforms for collaborative learning in health care, particularly since the COVID-19 pandemic, as they allow health care professionals to overcome barriers such as geography, time, and cost [27-29]. VCoPs have been shown to alleviate professional isolation, improve retention, foster interprofessional collaboration, and provide a risk-free environment for active participation [30,31]. Despite the potential benefits, only a few studies, including our Community Fracture Capture (CFC) Learning Hub pilot [32], have explored the role of internet-based learning activities in addressing the osteoporosis care crisis, maximizing health care resources, and delivering accessible skeletal health care at reduced costs [33,34].

Given the potential of VCoPs to offer a flexible and accessible way to enhance PCPs’ knowledge and confidence in osteoporosis management, and the current lack of studies evaluating their effectiveness, this study is guided by the following research question: “How do PCPs engage with a VCoP to build knowledge and confidence in osteoporosis care?” and “How can their engagement be improved?” This study will address the research question by evaluating PCPs’ engagement with an innovative VCoP, the CFC Learning Hub.

Our CFC Learning Hub served as an interactive, case-based, small-group online learning platform with flexible, tailored modules designed to address the fracture treatment gap in the community, promote current bone health practices for PCPs, and support the expansion of the e-learning hub and fracture liaison models at the primary care level, both in Australia and globally [35]. The quantitative evaluation of the program provided strong positive feedback, with 82% of participants reporting satisfaction with the program content and 89% indicating that they were likely to recommend it to their colleagues, suggesting that it is tailored to address the challenges faced by PCPs and could also be relevant in diverse health-related and other fields [36]. A qualitative analysis of the CFC model offers valuable insights into how learners interact with and experience such a novel learning model, complementing quantitative findings by adding context and depth to the interpretation of observed outcomes.

#### Qualitative Evaluation of the CFC Model

In addition to the insights gained from the quantitative analysis of the CFC Learning Hub, a qualitative evaluation of this innovative tool provides crucial information to assess the effectiveness of the model in delivering professional training to Australian PCPs and helping to bridge gaps in community-based fracture treatment. Qualitative inquiry explores the “how” and “why” behind decision-making, providing an in-depth and personalized understanding of PCPs’ behaviors, experiences, and real-world environments [37]. For instance, a recent study used qualitative analysis to investigate health practitioners’ perceptions of web-based learning to support patient behavior change [38]. Our study aims to identify themes related to barriers and facilitators of online learning, PCPs’ perceptions of the program, and challenges in osteoporosis management in general practice. We propose that the CFC Learning Hub can serve as a peer-to-peer platform providing up-to-date fracture prevention knowledge to PCPs of diverse backgrounds and experience levels, while also enhancing their confidence and motivation in osteoporosis care, as measured using analytics tools.

### Objectives

The objectives of the study are to identify (1) themes related to barriers and facilitators to PCPs’ interactions with the CFC Learning Hub online learning tool and (2) PCPs’ perceptions of the program.

## Methods

### Methodology

The CFC Learning Hub is a secure, flexible, internet-based digital platform—accessible via a web browser or digital application—designed to foster a collaborative learning environment. It features an interactive discussion forum where users can share their own case studies and take part in structured discussions led by bone specialists and experienced PCP moderators to meet their educational objectives. Additionally, the hub contains a comprehensive knowledge base, allows participants to submit questions and feedback, and includes weekly interactive quizzes to encourage active participation. The platform uses online surveys and backend analytics to assess initial knowledge, track participants’ activity, and evaluate progress, providing important insights into the learning journey and improvements after the course is completed. Participant feedback was used to inform ongoing review and refinement of the program after each cycle. The complete protocol for CFC Learning Hub design and implementation is available in our published protocol paper [35].

### Participant Recruitment

PCP participants in the CFC Learning Hub were recruited through the Praxhub platform, a leading online resource for health care professionals’ continuing education [39]. Invitations were distributed through professional bodies and Praxhub’s internal registry. Interested PCPs were contacted by the project manager, screened for eligibility, and enrolled if they met the criteria, consented to participate in the 6-week program, and provided electronic consent for the use of deidentified data. Recruitment took place from May 2022 to August 2023. Participants were invited to submit anonymized case studies from their own practice, which were discussed throughout the program to support engagement and clinical relevance. The platform’s private group setting (12-16 PCPs/cycle) was chosen to foster a supportive and engaged learning environment, with participants receiving weekly modules, access to discussions, and interactive quizzes, all within a secure and controlled space to encourage active participation.

### Participants

Between May 2022 and October 2023, the hub conducted four 6-week small-group cycles, involving 55 PCPs in total. Of these, 35 (64%) participants actively took part in the end-of-cycle evaluation, and 33 (60%) participants engaged with the activities by posting comments or queries. Among the participants, 44 (80%) practiced in metropolitan areas, the median (IQR) years of practice was 22 (16-34) years, and 11 (31%) participants reported some difficulties using the platform [36]. Throughout the cycles, 8 PCPs and specialists served as moderators, facilitating the activities and guiding discussions. All moderators contributed actively by posting or commenting on ongoing discussions.

### Qualitative Data

The qualitative data for the project were collected from two sources: (1) comments on weekly discussions provided by participants and moderators and (2) participants’ responses to four qualitative questions in the end-of-cycle evaluation questionnaire (the questionnaire consisted of 15 questions, including 4 qualitative open-ended questions analyzed in this paper and 11 multiple-choice closed-ended questions used for quantitative analysis [Ahmed M Fathalla, PhD, unpublished data, June 2025]). The questions were: “If you experienced difficulties using the platform, what were they?” “What improvements could be made to the platform to enhance your experience in the program?” “What improvements could be made to the program to enhance your overall experience?” and “What information, content, or functions would motivate you to return to the CFC Learning Hub for learning purposes in the future?” Both the discussion comments and responses to the qualitative questions were compiled for qualitative analysis.

### Qualitative Analysis

To achieve the study’s aims, relational content analysis was undertaken, guided by Braun and Clarke [40,41]. Specifically, 2 researchers independently identified codes in the first instance. In this initial step, researchers used relational content analysis and used both semantic (capturing explicit and obvious meanings of the text) and latent (capturing tone, intention, and underlying assumptions) inductive, bottom-up coding approaches to explore a wider range of codes. In the second phase, guided by the research questions of the project, the 2 researchers engaged in rigorous discussion and debated the codes, using both inductive and deductive approaches, to assess patterns that coalesced around key themes and collaboratively refined and defined themes and subthemes from the qualitative data. Finally, all data were checked against the list of subthemes to explore the representation of each subtheme in the dataset by a single researcher, with a random sample validated by a senior researcher.

Due to the innovative nature of the e-learning hub, themes were created from participants’ transcripts after each cycle and validated through a rigorous evaluation of past and current initiatives in this area in the literature [17,29,34]. As the program progressed, appropriate adjustments were made to the design and content of the e-learning hub based on the feedback.

The relational content analysis was undertaken [42,43]. The results of the thematic analysis are reported using both top-down and bottom-up approaches, clustering a wide range of ideas together, followed by designating the themes and subthemes that emerged. All results are supported by exemplary and representative quotes provided by participants.

### Ethical Considerations

The Melbourne Health Human Research Ethics Committee approved this project (site reference no 2016.24). Electronic consent was obtained from PCP participants for the use of their deidentified data in research and auditing. The consent process, including the waiver of consent for case study patients whose anonymized information was used, was facilitated through Praxhub’s tools for data collection and management. No financial compensation was provided to participants in this program.

## Results

### Overview

This section presents data from the postactivity feedback survey responses of participating PCPs and comments posted by PCPs on the module discussion threads.

### PCPs-CFC Learning Hub Interaction

This section reports on the PCPs’ interactions with the program and their feedback on the experience, which can be broken down into 4 main themes: user experience–guided platform design, learning-supportive course structure, learners’ different styles and preferences, and program content.

An overview of the subthemes’ definitions and their distribution throughout the cycles is provided in Table 1.

**Table 1.** Qualitative analysis of primary care physicians’ interactions with the Community Fracture Capture (CFC) Learning Hub, including 4 themes and their subthemes.

Theme and subtheme	Definitions	Subtheme was present in the following cycles
User Experience–Guided Platform Design		
Flow Between Different Components of The Program	The ease and clarity with which users can navigate and transition between different sections and features within the program’s interface.	Cycles 1 and 2
Minimization of Separated Sections Within the Learning Platform	The integration of content and the reduction of fragmented areas within the platform.	Cycles 1 and 2
Clarity of navigation options on the platform	The visibility and intuitiveness of navigation tools and pathways within the platform.	Cycles 1, 2, and 3
Learning-Supportive Course Structure		
Explicit Easy-to-Access Linking Between Case Studies and Foundation Knowledge Papers	The clear and direct integration of theoretical content with practical case studies.	Cycles 1, 2, and 4
Helpful Weekly Summary of Essential Learnings	The inclusion of concise, accessible summaries that highlight key learning points, expectations, and reference tools on a weekly basis.	Cycles 1, 2, and 4
Quizzes that support Ongoing Learning	The use of quizzes is designed to reinforce learning, assess knowledge, and support ongoing retention of course material.	Cycles 1 and 4
Learners’ Different Styles and Preferences		
Webinars Support Learners Preferring Synchronous Interactions	A synchronous communication learning format that includes live, scheduled sessions and enables real-time interaction, discussion, and engagement.	Cycles 2, 3, and 4
Asynchronous Program Structure Supports Learners preferring Self-Study	A flexible, self-paced learning format that allows participants to engage with content independently.	Cycles 2 and 4
Some Learners prefer Didactic Sessions	A preference for structured, instructor-led content delivery.	Cycles 1 and 4
Significance of Peer-to-Peer Learning Continues	The ongoing value is placed on collaborative learning through the sharing of experiences, insights, and practical strategies among peers within the learning environment.	Cycles 1, 2, and 4
Online Discussions Enhanced Learning for Interaction-Seeking Learners	The role of interactive online discussions in enriching the learning experience for participants who value peer-to-peer engagement and collaborative dialogue.	Cycles 1, 2, 3, and 4
Active Discussion Participation Evidenced by More Questions	High levels of learner engagement are demonstrated through frequent questioning.	Cycles 1, 2, 3, and 4
Program Content		
Topic Suggestions	Participant-identified areas of interest or need for additional content.	Cycles 1, 3, and 4
Participant Suggestions for Reinforcement and Long-Term Knowledge Retention	Learner preferences for ongoing opportunities to revisit and reinforce educational content.	Cycles 1 and 2

The presence and progression of subthemes across the program cycles reveal both consistency and improvement in the program’s development. For instance, challenges related to navigation and fragmented sections were noted in cycles 1 and 2, but these subthemes did not appear in later cycles, suggesting enhancements to platform integration and user experience over time. Similarly, the increasing emphasis on responding to diverse learning preferences is reflected in the increased recognition of both synchronous and asynchronous learning features from cycle 2 onward. While some learning-supportive elements, such as quizzes, appeared only in cycles 1 and 4, their recurrence may indicate targeted revisions based on participant feedback. The consistent presence of peer-to-peer interaction subthemes across all cycles underscores their continued value, while the disappearance of certain early concerns suggests that the program has evolved in direct response to user needs. Overall, the themes reflect the improvements made in each cycle in response to participants’ feedback.

### User Experience–Guided Platform Design

#### Overview

Participants stressed that a well-designed online learning platform is essential for improving their user experience, especially given their limited time. They noted that features such as intuitive navigation, easy access to resources, and responsive support are crucial. They also mentioned that minimizing complexity and ensuring key information is accessible helps them engage effectively with the content, thereby supporting their professional development. Based on the data collected for this theme, this theme was further divided into 3 subthemes.

#### Flow Between Different Components of the Program

Some participants mentioned encountering challenges with interface design. The issues reported included difficulty in finding specific information among a vast amount of reference material, leading to time consumption, and confusion about the setup of tiles under the education tab and the main page. While acknowledging that the course materials were helpful, they highlighted that the navigation and organization of information were not as streamlined or intuitive as needed, pointing to a need for improved clarity and usability in the system’s design to enhance user efficiency and reduce the learning curve.

One participant from cycle 1 mentioned:

*Going in and out of the hot topic to check different references was time consuming as some of the answers were difficult to find in amongst the vast amount of reference material.*

Another participant from cycle 2 highlighted facing problems with flow between the program components:

*Issues with going between case study and discussion. Confusing set up of tiles under education and also on the main page. It took me awhile to realise that the tiles were under the education tab*.

It is notable that after the initial cycles, improvements such as the provision of more detailed and advanced platform induction and changes to content layout were made to ensure an improved user experience. In the following cycles (cycles 3 and 4), no similar comments regarding the need for improvements were made.

#### Minimization of Separated Sections Within the Learning Platform

Many participating PCPs indicated that minimizing separated sections within the online learning platform significantly enhances their user experience by creating a more cohesive and streamlined learning environment. They noted that when content is organized in a unified manner rather than fragmented across multiple sections, learners can more easily navigate through materials and maintain focus. According to them, this integration reduces cognitive load, allowing users to connect concepts more effectively and progress more smoothly through their courses. They reported feeling frustrated and disoriented, struggling to connect concepts and maintain their focus when navigating through fragmented and disjointed sections.

One participant of cycle 2 mentioned:

Some materials can only be found with one particular way of entering the platform. The same material gets “lost” and hard to go back to if one “strayed” to a different interface, for instance, at one moment I was viewing week 5 communications/discussions, then I went to week 6. Week 5 material subsequently "lost" and very hard to get back in.

Another cycle 2 participant suggested that reducing separated sections would enhance the learning experience:

More cases would have been better with simple reading material incorporated into learning tiles.

Similar to the “Flow Between Different Components of the Program’ subtheme, no comments regarding section separation were made in cycles 3 and 4. This followed changes to the program format that reduced content separation and included a detailed navigation session in the Week 1 webinar.

#### Clarity of Navigation Options on Platform

A considerable proportion of cycles 1, 2, and 3 suggested that having clear navigation options on an online learning platform enhances their overall user experience. Many of them mentioned that clarity of navigation options allowed them to easily find and access various sections of the course, reducing frustration and saving time, and helped learners to focus on the material without getting lost or overwhelmed by the platform’s structure. Several participating PCPs highlighted that, with intuitive navigation, learners can quickly locate specific content, track their progress, and seamlessly transition between modules.

One cycle 1 participant expressed frustration over the unclear platform navigation options:

(I) *could not navigate easily across platform, could not easily access previous weeks’ materials unless used old links to access only them, site generally tedious.*

Another cycle 2 participant highlighted facing difficulties due to unclear navigation mapping throughout the platform:

For the full list of Tiles to be accessed you had to click on the education section which was confusing. It was difficult to navigate back to the cases.

As with the previous 2 subthemes, no comments regarding issues with unclear navigation options were received in cycles 3 and 4, following changes to the platform layout that provided a clearer map of the platform and the inclusion of a navigation session early in the program.

### Learning-Supportive Course Structure

#### Overview

Participants mentioned that a well-structured course that connects practice-based case studies with core papers can greatly improve learning. They also suggested that the provision of regular summaries can help to reinforce key information and prevent cognitive overload, while after-activity quizzes can serve to continuously assess and deepen program understanding. They noted that this integrated approach fosters a more coherent and impactful learning experience, facilitating both comprehension and retention of the material. This theme, based on the data collected, was further categorized into 3 subthemes.

#### Explicit Easy-to-Access Linking Between Case Studies and Foundational Knowledge Papers

Several participants emphasized the need for connections between case studies and foundational knowledge papers to be made explicit and easily accessible. They highlighted that clear, direct links between practical examples and theoretical content would enhance their understanding and application of the material. Additionally, they mentioned that ensuring these connections are readily accessible and well-defined would improve navigation and reinforce learning by helping participants link theoretical concepts to their application in real-world scenarios.

A cycle 1 participant suggested:

I think it would be better to merge the case study and hot topic into one tile. It wasn’t so easy to switch from one to the other.

A cycle 4 participant expressed confusion:

It was a bit confusion how to move on from one episode to another.

The observations from these quotes indicate a need for improved integration and navigation within the online learning platform. The feedback suggests that enhancing the course structure could improve user experience by making transitions between case studies and foundational knowledge papers smoother and more intuitive.

#### Helpful Weekly Summary of Essential Learnings

Some participants highlighted a consistent desire for clearer, more accessible summaries and highlights throughout the course. They expressed a need for concise summaries that clearly outline the knowledge and management expectations for participating PCPs, as well as key points and reference tools. They also suggested incorporating bullet-point summaries and providing weekly overviews or links to resources to better track and review the learning content.

A cycle 2 participant added:

The only thing I would add is to summarise and highlight what knowledge and level of management is expected of GPs. Sometimes I think that got lost in the discussion of complex issues and there is a huge amount of detail in this topic. I would like to have seen this summary at the end of the course.

A cycle 4 participant proposed:

Perhaps adding a summary of all the learning points in bullet style. And having a Summary of learning points generated by the discussions.

These suggestions point to a need for enhanced clarity and organization to ensure that crucial information is not lost in complex discussions and that learners can easily access and review essential material.

#### Quizzes That Support Ongoing Learning

Some members reported concerns about the quizzes used in the program, highlighting issues with their difficulty and alignment with the course material. They found the quizzes challenging, with questions that were hard to answer based on the provided resources.

A cycle 1 participant mentioned:

Quiz questions could be improved in educational validity.

A cycle 4 participant highlighted:

Some of the quizzes questions were too hard and it was difficult to find the answers, even though I read through all the resources.

Overall, these observations suggest a need for improving quiz design and content alignment to better match the course content and enhance learner engagement and assessment accuracy.

### Learners’ Different Styles and Preferences

#### Overview

While our program supported the learners in a less directive learning style, PCP participants in the CFC Learning Hub exhibited varying interests in diverse learning styles, such as synchronous interactions, self-study, didactic sessions, and online discussions. For those interested in different learning styles, the program aimed to accommodate varied learning approaches by offering a range of instructional methods. The program supported real-time engagement through live sessions, provided flexibility for independent study, incorporated structured didactic content for foundational knowledge, and fostered collaborative learning through interactive discussions. Additionally, in later cycles (ie, cycles 3 and 4), prerecorded short video discussions were included in each week of the cycle to engage learners preferring one-way direction education. Further, we updated our communications with content that encouraged participation in other learning styles, such as peer-to-peer discussion. This approach aimed to ensure that learners could engage with the material in ways that aligned with their individual learning styles, enhancing overall effectiveness and satisfaction. However, it is important to address that there is no one-size-fits-all approach to learning styles, as various methods will appeal differently to various learners.

The learners’ different styles and preference themes were further broken down into 6 subthemes.

#### Webinars Support Learners Preferring Synchronous Interactions

Some members of the program expressed their preference for live, interactive elements in online learning. Specifically, they expressed their desire for regularly scheduled virtual meetings or webinars that facilitate real-time discussions, enable immediate question-and-answer sessions, and enhance their engagement. This cohort of participants believed that live interactions, as opposed to asynchronous messaging or comment posting, foster more effective and dynamic conversations. According to their perspectives, live webinars with interactive features, such as live chat and question-and-answer, tend to generate greater participation and maintain interest better than asynchronous communication methods.

A cycle 2 participant mentioned:

I would have liked a Zoom meeting every week at a scheduled time to discuss any questions with your fellow hub members. I would have preferred a live discussion rather than messaging.

Also, a cycle 4 member commented:

In my experience I feel like there is more participation during a live webinar with a Q&A session as it is more like a conversation, whereas the posting of comments during the whole week, people logging in at different times, perhaps losing interest, feeling intimidated and so on isn’t as effective in generating conversations.

Overall, these learners expressed their inclination toward incorporating more video and live discussion formats to improve their learning experience.

#### Asynchronous Program Structure Supports Learners Preferring Self-Study

Several members highlighted positive aspects of the program experience and its support for a self-study learning style. Participants demonstrated an impression with the quality of feedback provided by both PCP and specialist moderators, suggesting that the level of support and interaction was highly valued. They also appreciated the overall style and structure of the course, finding it enjoyable and effective.

A cycle 2 participant said:

I was Impressed with the level of feedback by the main facilitator and by the specialist involved.

A cycle 4 member mentioned:

I did enjoy the way you could back and forth between the different "episodes " of the 6 in this series. Ensure(s) it is easy for a GP to access all 6 sessions.

According to this cohort of CFC Learning Hub members, the learning style providing flexibility to navigate between different episodes or sessions was noted as a strong feature, ensuring easy access for learners.

#### Some Learners Prefer Didactic Sessions

Some participants preferred a more didactic and directive learning experience. They desired a consistent instructional approach, with a clear progression from didactic lectures to case presentations and interactive sessions, rather than self-studying the provided resources. Additionally, they asked for an increased use of structured and concise video discussions, avoiding open-ended conversations that can disrupt the flow of their learning.

A cycle 1 participant mentioned:

I need a proper lecture in each lesson of the week instead of sending the resources only. The change to didactic lecture then case presentation then interactive session among the participants.

A cycle 4 participant recommended:

Increase video discussions with specialists. Not have the longwinded discussions that loses train of thought.

In summary, these observations highlight a desire among some members for a more structured learning environment with well-organized content delivery and focused discussions.

#### Significance of Peer-to-Peer Learning Continues

Some CFC members highly valued the exchange of diverse perspectives, practical suggestions, and collaborative discussions. They appreciated hearing various management viewpoints, acknowledged the practical advice on clinical practice, and expressed appreciation for the knowledge shared by moderators and colleagues. Additionally, they gave positive feedback on engaging and helpful discussions, indicating that the collaborative and interactive elements of the learning experience are particularly beneficial.

A cycle 1 participant mentioned:

It is good to hear other people views in management in their practices. Thanks everyone for their input.

In another comment, the same participant mentioned:

I also wanted to thank you all the facilitators, presenters, and my fellow colleagues for sharing your knowledge. Many thanks for discussing the case studies we sent which was immensely helpful.

Overall, the observations reveal that some participants valued the exchange of diverse viewpoints, appreciated practical suggestions and expert input, and responded positively to collaborative discussions.

#### Online Discussions Enhanced Learning for Interaction-Seeking Learners

Learners preferring interactions reflected a strong appreciation for the educational content, effective communication, and supportive interactions provided throughout the learning sessions. Those participants frequently expressed gratitude for the helpful and engaging nature of the educational materials, case study discussions, and presentations. There was also recognition of the value of the collaborative efforts of moderators, specialists, and the IT team in making the sessions informative and engaging. Many specifically commended moderators for their comprehensive answers, concise presentations, and overall contributions, noting that these addressed their questions and enhanced their learning experience.

A cycle 2 participant expressed gratitude:

Thanks (moderator name) for answering my copious questions. I’m so glad for this learning activity. I’ve learned a lot but there is so much more to learn. It’s so challenging for GPs to keep up with every discipline. In recent weeks I’ve been reviewing osteoporosis management of many of my patients…hoping to do a better job! I’m very appreciative of all the educators’ contribution & comments from everyone in this group. Thank you!!

One cycle 4 participant commented:

Just a wee word to thank the facilitators, specialists and it crew for a fantastic approachable interesting formative expose on osteoporosis. Well done all of you…thanks for all the answers to my questions…best wishes

In summary, feedback from these interaction-seeking learners highlights a high level of satisfaction with the educational activity and a deep appreciation for the support and knowledge shared.

#### Active Discussion Participation Evidenced by More Questions

Many participants highlighted a preference for interactive questioning and active dialogue as key components of their learning process, demonstrated by asking more questions. Additionally, when participants asked questions, they contributed to a collaborative environment in which knowledge was coconstructed through shared inquiry and discussion, as their questions encouraged other members and moderators to engage interactively and contribute further to the conversation.

Below are some examples of questions asked by engaged participants throughout the program’s different cycles.

A cycle 1 participant commented and asked:

If patient has calcium rich diet, I tend not to prescribe ca supplements but in frail elderly patients the calcium intake is usually insufficient, so supplements are indicated. I do not usually prescribe larger less frequent dosing of vitamin D since I think compliance may be more of a problem, though I have no sound evidence for that opinion. what do other people think?

A cycle 2 participant asked:

Thanks (name) for such a thorough work up of this very complex case. Would appreciate your comment on my pt. 81 woman new to my practice ….How do you monitor response? Would it be based on no further fractures or T scores not dropping Given her age, would you lean towards denosumab indefinitely? Another question we didn't get to answer from previous hot topic was when to check testosterone level in osteoporosis cases eg Excessive ETOH intake, medications like steroids, anticonvulsants.

This dynamic of asking questions fostered a richer, more interactive learning experience, enhancing both individual comprehension and collective knowledge.

### Program Content

#### Overview

Some participants shared their thoughts on the program content, offering valuable feedback that can lead to actionable improvements. Their reflections provide useful insights into how the content may be enhanced in future cycles and how the program may continue to support members even after the cycle ends. The program content is further divided into 2 subthemes: Topic Suggestions and Reinforcement and Long-Term Knowledge Retention.

#### Topic Suggestions

Participants across all cycles suggested a range of additional topics to include in the program, highlighting a desire for both broad and detailed topics. These included practical topics such as managing common day-to-day PCP presentations, understanding government-subsidized vs nonsubsidized treatment approaches under national health care reimbursement schemes (eg, Australia’s Pharmaceutical Benefits Scheme and Medicare Benefits Schedule), and incorporating content aimed at preventing morbidity and mortality. Participants also expressed interest in more detailed clinical content, including management across different case scenarios, selection of drugs in osteoporosis treatment, and recent breakthroughs in the field. Additionally, suggestions were made to expand the scope of the program to include women’s health, pediatrics, and information on menopausal hormone therapy and premenopausal osteoporosis.

In response to this feedback, later program cycles were adapted to include more case-based discussions related to suggested topics, along with more detailed treatment guidance. Broader topics such as women’s health and pediatrics were acknowledged as valuable and noted for future inclusion beyond the current scope of the program.

In summary, this subtheme suggests a need for the program to incorporate a diverse array of topics, addressing both practical concerns and emerging advances to better meet participants’ varied learning needs.

#### Participant Suggestions for Reinforcement and Long-Term Knowledge Retention

Several participants in the program valued opportunities for ongoing learning and reinforcement of the program material. Many expressed a desire for periodic refreshers or updates to help retain and apply their knowledge over time. Suggestions included offering brief annual refresher programs, updates on osteoporosis, and the ability to revisit core content. Some participants specifically noted the need to review studies cited in the educational material and to have continued access to resources such as the national health care reimbursement schemes, including Pharmaceutical Benefits Scheme eligibility criteria, acknowledging the challenge of recalling detailed information after the initial delivery of the program.

These observations suggest that incorporating regular review sessions and updates into the program could enhance its effectiveness and support long-term learning for participants.

## Discussion

### Overview

The CFC Learning Hub demonstrated its potential as a virtual community of practice for bridging osteoporosis care gaps in primary care. Our qualitative analysis revealed critical insights into platform usability and learning dynamics, aligning with broader literature on VCoPs and online professional development.

### Principal Findings

This qualitative analysis of the CFC Learning Hub revealed that platform usability challenges (eg, fragmented navigation) were resolved through iterative design, enhancing engagement in later cycles. Case-based learning, coupled with foundational papers, fostered clinical reasoning; however, quiz-resource misalignment was reported as a hindrance to the assessment process. The analysis also showed that diverse learning preferences—including synchronous webinars and asynchronous peer- and specialist-supported self-study—were successfully accommodated, with peer-to-peer and PCP-to-specialist discussions driving collaborative problem-solving. In summary, these findings indicate that the CFC Learning Hub effectively supported engagement, learning, and collaboration while highlighting areas for continued refinement.

### Platform Design and Usability

CFC Learning Hub participants prioritized intuitive navigation and integrated content delivery, aligning with studies highlighting usability as a cornerstone of effective e-learning platforms [31,44-48]. Challenges with fragmented sections (eg, difficulty locating materials between weeks) in early cycles mirror findings by Barnett et al [31], who identified disjointed interfaces as barriers to engagement in VCoPs. Iterative improvements, such as streamlined layouts and navigation guides, resolved these issues in later cycles, demonstrating the value of agile design principles in online learning [49,50]. These adjustments align with user experience–guided design digital health care capability frameworks [51-54], which prioritize learner feedback in platform optimization.

### Case-Based Learning and Formative Assessment

Participants valued case studies, particularly when linked to foundational knowledge papers (eg, discussions on osteoporosis management in older adults). This parallels evidence that case-based learning catalyzes member engagement and enhances the learning experience [55,56]. The integration of case studies with foundational knowledge and weekly summaries reflects established best practices in adult learning theory, which prioritize contextual and scaffolded content delivery [24]. Participants’ requests for concise summaries and quizzes aligned with course materials echo findings by Burgess et al [57], who demonstrated that structured summaries improve retention in medical education. However, mismatches between quiz difficulty and provided resources (eg, participants struggling to locate answers) may indicate a gap in formative assessment design—a gap noted in studies of online continuing professional development [25]. Future iterations of the CFC Learning Hub could benefit from adopting spaced repetition tools, such as weekly summaries and modular content delivery, to mitigate cognitive overload, aligning with studies on the application of spaced learning in health care professional education [58-60].

### Accommodating Diverse Learning Preferences

The coexistence of synchronous (live webinars) and asynchronous peer- and specialist-supported (weekly self-paced modules) engagement underscored the heterogeneity of learner needs—a finding consistent with observations in several studies that investigated the use of VCoPs by health care professionals, including our CFC Learning Hub [32,61]. While some VCoP participants thrived in live interactions, others preferred reflective, self-paced learning, offering a “polycontextual” environment suitable for learners with diverse learning preferences [62]. The integration of prerecorded videos in later cycles addressed this diversity, supporting multimodal learning theory, which asserts that diverse content delivery enhances accessibility [63]. This adaptability is critical in primary care, where time is identified as a significant barrier for participation in VCoPs [64,65].

### Implications for Osteoporosis Management

In terms of clinical implications, the findings from this study suggest that the CFC Learning Hub’s unique structure can play a critical role in improving health care professionals’ understanding of osteoporosis management. By discussing the latest research, sharing clinical guidelines, and encouraging critical reflection on practice, the hub helped participants stay informed and equipped to manage osteoporosis more effectively. This approach is crucial in osteoporosis management, where evolving research and guidelines require practitioners to stay up-to-date with the best practices [66]. Participating in the CFC Learning Hub can facilitate knowledge transfer by enabling participants to observe how the acquired knowledge can be applied within their clinical practice [67].

Moreover, the collaborative nature of the discussions allowed participants to learn from one another, making the learning process more relevant and applicable to their own practices, assisting in the construction of new relationships and reinforcing existing ones, thereby creating a sense of community [68]. The CFC Learning Hub fostered a collaborative, positive, and supportive environment, which plays a key role in helping participants feel comfortable sharing with the community, interacting with other members, and reducing professional isolation [31,62,68]. This type of professional development is particularly beneficial in supporting the management of complex conditions such as osteoporosis, where tailored, patient-centered care is essential [69]. The opportunity to discuss challenging cases and share clinical strategies can help practitioners refine their approaches and improve patient outcomes.

### Limitations and Future Research

While this study provides valuable insights into the role of moderated online learning environments, there are some limitations to consider. First, self-selection bias may have influenced participation, as PCPs motivated to improve osteoporosis care were likely overrepresented. Second, participants were recruited through the Praxhub platform, with most being practitioners located in metropolitan areas and having more than 10 years of experience, which may limit the broader applicability of the findings. As a result, the results might not fully reflect the impact of the training across a wider community. Further evaluation of the program’s benefits is necessary, specifically targeting regional general practitioner practices and/or less experienced practitioners. Moreover, as a limitation of qualitative studies, the study relied on self-reported data, which may introduce biases in participants’ responses. However, we aimed to mitigate this limitation by using a rigorous, peer-reviewed analysis protocol recognized for its reliability and validity [42,43]. Future research could explore the effectiveness of these learning strategies in larger and more diverse health care settings, using objective measures of clinical outcomes to assess the impact of such programs on patient care.

Further studies could also investigate the long-term effects of online learning programs on clinical practice. While this study demonstrates immediate engagement and knowledge sharing, it remains unclear whether these learning gains translate into sustained changes in clinical behavior and patient outcomes. Longitudinal studies could help determine whether the knowledge and skills gained through these programs are retained over time and lead to improved patient management.

### Conclusion

The CFC Learning Hub exemplifies the potential of VCoPs to transform osteoporosis care by creating interactive and participant-centered learning environments for health care professionals. Its iterative design and emphasis on peer collaboration address systemic barriers while accommodating diverse learning needs. By addressing usability barriers, accommodating diverse learning styles, and leveraging moderator expertise, the platform fosters meaningful engagement in osteoporosis management. As osteoporosis burdens rise with aging populations, tools such as the CFC Learning Hub will be critical in equipping PCPs to close the gap.

## Abbreviations

CFC: Community Fracture Capture
DXA: dual-energy x-ray absorptiometry
FLS: fracture liaison service
PCP: primary care physician
VCoPs: Virtual Communities of Practice
